# Increased 3-hydroxypropionic acid production from glycerol, by modification of central metabolism in *Escherichia coli*

**DOI:** 10.1186/1475-2859-13-64

**Published:** 2014-05-07

**Authors:** Kento Tokuyama, Satoshi Ohno, Katsunori Yoshikawa, Takashi Hirasawa, Shotaro Tanaka, Chikara Furusawa, Hiroshi Shimizu

**Affiliations:** 1 Department of Bioinformatic Engineering, Graduate School of Information Science and Technology, Osaka University, 1-5 Yamadaoka, Suita, Osaka 565-0871, Japan; 2 Department of Bioengineering, Tokyo Institute of Technology, 4259 Nagatsuta-cho, Midori-ku, Yokohama 226-8501, Japan; 3 Quantitative Biology Center, RIKEN, 6-2-3 Furuedai, Suita, Osaka 565-0874, Japan

**Keywords:** 3-hydroxypropionic acid, *Escherichia coli*, Glycerol, Genome-scale metabolic model, Central metabolism

## Abstract

**Background:**

3-hydroxypropionic acid (3HP) is an important chemical precursor for the production of bioplastics. Microbial production of 3HP from glycerol has previously been developed through the optimization of culture conditions and the 3HP biosynthesis pathway. In this study, a novel strategy for improving 3HP production in *Escherichia coli* was investigated by the modification of central metabolism based on a genome-scale metabolic model and experimental validation.

**Results:**

Metabolic simulation identified the double knockout of *tpiA* and *zwf* as a candidate for improving 3HP production. A 3HP-producing strain was constructed by the expression of glycerol dehydratase and aldehyde dehydrogenase. The double knockout of *tpiA* and *zwf* increased the percentage carbon-molar yield (C-mol%) of 3HP on consumed glycerol 4.4-fold (20.1 ± 9.2 C-mol%), compared to the parental strain. Increased extracellular methylglyoxal concentrations in the Δ*tpiA* Δ*zwf* strain indicated that glycerol catabolism was occurring through the methylglyoxal pathway, which converts dihydroxyacetone phosphate to pyruvate, as predicted by the metabolic model. Since the Δ*tpiA* Δ*zwf* strain produced abundant 1,3-propanediol as a major byproduct (37.7 ± 13.2 C-mol%), *yqhD*, which encodes an enzyme involved in the production of 1,3-propanediol, was disrupted in the Δ*tpiA* Δ*zwf* strain. The 3HP yield of the Δ*tpiA* Δ*zwf* Δ*yqhD* strain (33.9 ± 1.2 C-mol%) was increased 1.7-fold further compared to the Δ*tpiA* Δ*zwf* strain and by 7.4-fold compared to the parental strain.

**Conclusion:**

This study successfully increased 3HP production by 7.4-fold in the Δ*tpiA* Δ*zwf* Δ*yqhD E. coli* strain by the modification of the central metabolism, based on metabolic simulation and experimental validation of engineered strains.

## Background

3-hydroxypropionic acid (3HP) has recently attracted attention due to its availability as a precursor of valuable chemicals such as acrylic acid, β-propiolactone, and malonic acid [1,2]. Its polymerized form, poly(3HP), is a promising alternative to petrochemical-derived plastic [3,4]. Because of this superior industrial availability, 3HP was designated as one of the top value-added chemicals produced by biomass, by the U.S. Department of Energy [5,6]. In the commercial bioproduction process, the substrate has a significant impact on production cost. Glycerol is a potential substrate for bioproduction considering that the recent expansion of biodiesel production has caused a surplus of glycerol as its byproduct, and a subsequent decrease in the price of glycerol [7-9].

The microbial production of 3HP from glycerol has been developed using a natural 3HP producer, *Klebsiella pneumoniae*[10-13]. Expression of the heterologous glycerol dehydratase and aldehyde dehydrogenase enabled 3HP to be produced in the non-natural 3HP producers *Pseudomonas denitrificans*[14,15] and *Escherichia coli*[16-19]. To date, various studies have reported increased 3HP production [10-19]. For example, Rathnasingh et al. optimized the expression level of each enzyme in this pathway in *E. coli*[17]. Ashok et al. deleted *dhaT* and *yqhD* to reduce the production of byproducts in *K. pneumoniae*[12]. Kim et al. modified glycerol metabolism in 3HP-producing *E. coli* and developed fed-batch cultivation with simultaneous feeding of glycerol and glucose [19]. As described above, most previous studies focused on the optimization of metabolic reactions from glycerol to 3HP and culture conditions. Considering the whole metabolic network, modification of other pathways, as well as the biosynthetic pathway of the target product, is also a key strategy for increasing the metabolic flux, leading to enhanced target production. For example, target production can be enhanced by improving the redox balance and reducing byproduct formation via gene knockout and overexpression [12,20].

Recently, *in silico* metabolic simulation has been developed to consider whole metabolic networks. A genome-scale metabolic model, which includes most of the metabolic reactions of the cell [21-23], can estimate the flux distribution of the whole metabolic network using flux balance analysis (FBA) [24,25] by assuming the steady states of metabolic reactions and maximizing objective functions such as cell growth [26,27]. This method can be used to simulate the effects of gene modifications on target production and identify candidate genes for metabolic engineering [28-30]. Successful improvements in target production have been reported in many such simulations [31-33].

The introduction of heterologous genes and gene manipulations does not always produce the expected growth behavior and target production in engineered strains. Experimental validation of engineered strains provides useful information about the actual metabolic state of a cell such as bottleneck reactions, growth inhibition factors, and redox imbalance [34-36]. A strain engineered on the basis of metabolic simulation should be evaluated experimentally to develop the next strategy for improving target production.

The current study aimed to enhance 3HP production from glycerol in *E. coli* by focusing on the whole metabolic network. Metabolic pathway modification was designed by integrating *in silico* gene-knockout simulation with the experimental validation of engineered strains. The genome-scale metabolic model of *E. coli* was used to design a metabolic network for increased 3HP production. Metabolic simulation identified two candidate genes to be deleted, *tpiA* and *zwf*, which are involved in central metabolism. Based on the simulation, knockout strains were constructed and 3HP production was successfully increased, as predicted. In addition, a gene, *yqhD*, related to the biosynthesis pathway of a major byproduct, 1,3-propanediol (1,3-PDO), was deleted to further increase 3HP production.

## Results and discussion

### Construction of a 3HP-producing strain in E. coli

**Figure 1:**
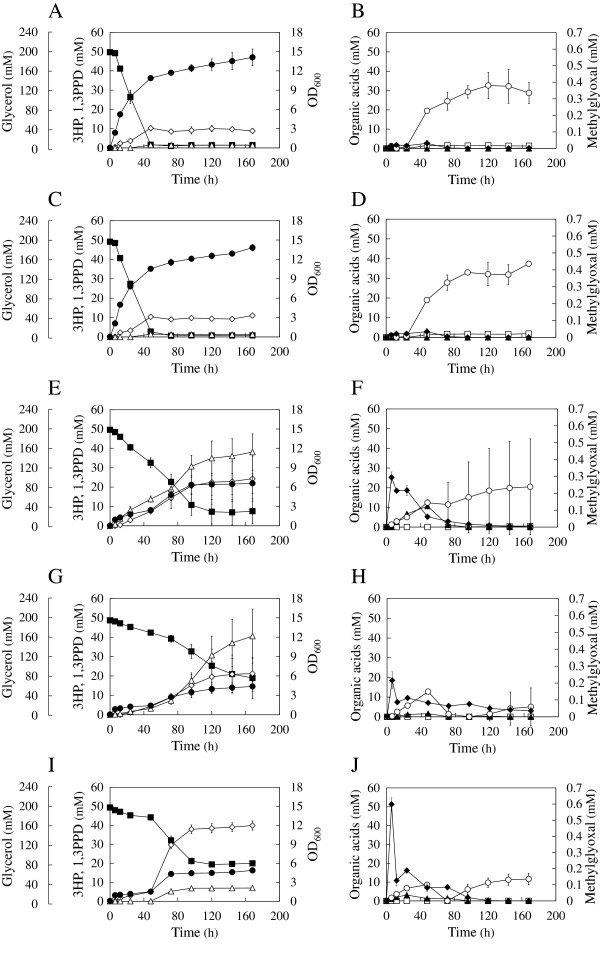
**Culture results of the 3HP-producing strains.** The culture results of the strains TK52 **(A, B)**, TK52z **(C, D)**, TK52t **(E, F)**, TK52tz **(G, H)**, and TK52tzy **(I, J)** are shown. Open diamond, 3HP; closed square, glycerol; open triangle, 1,3-PDO; closed circle, OD_600_; open circle, acetate; open square, succinate; closed triangle, lactate; closed diamond, methylglyoxal. Error bars represent standard deviation of triplicate experiments in TK52 and TK52z strains, and of nine replicate experiments in other strains. Some of the error bars are smaller than the symbols.

The 3HP biosynthetic pathway from glycerol consists of two reactions: the dehydration of glycerol to 3-hydroxypropionaldehyde (3HPA), catalyzed by glycerol dehydratase, and the oxidation of 3HPA to 3HP, catalyzed by aldehyde dehydrogenase [16]*.* Since *E. coli* does not possess the 3HP biosynthetic pathway, the 3HP-producing strain (TK52) was constructed by the overexpression of *dhaB* and *gdrAB*, which encode for glycerol dehydratase and glycerol dehydratase reactivase (from *K. pneumoniae*), respectively, and *aldH*, which encodes for aldehyde dehydrogenase (from *E. coli*), as described in a previous study [17]. TK52 was cultivated in M9 medium in a Sakaguchi flask and 3HP was produced at 4.6 ± 0.8% carbon-molar yield of 3HP on consumed glycerol (C-mol%) (Figure 1A–B and Table 1). Acetate was produced as a major byproduct (9.9 ± 1.8 C-mol%) and small yields of 1,3-PDO (0.8 ± 0.4 C-mol%) and succinate (0.9 ± 0.1 C-mol%) were also produced. Ethanol and formate were not detected in this strain and other strains that were constructed. Although *E. coli* does not possess 1,3-PDO biosynthetic pathways, the introduction of *dhaB* for 3HP production enabled production of 1,3-PDO as follows: glycerol dehydratase converts glycerol to 3HPA, and an endogenous alcohol dehydrogenase (encoded for by *yqhD*) further converts 3HPA to 1,3-PDO [13,32].

**Table 1:** Summary of the experimental results

	**TK52**	**TK52z**	**TK52t**	**TK52tz**	**TK52tzy**	Simulation^*4^ **iAF1260-3HP**	Simulation^*4^ ***ΔtpiA Δzwf***
Specific growth rate (1/h)^*1^	0.73 ± 0.00	0.71 ± 0.00	1st: 0.55 ± 0.01	1st: 0.54 ± 0.01	1st: 0.56 ± 0.01		
			2nd: 0.03 ± 0.01	2nd: 0.03 ± 0.00	2nd: 0.04 ± 0.01		
Maximum 3HP production rate (mmol/(g DC●h))^*2^	0.08 ± 0.02	0.09 ± 0.01	0.22 ± 0.14	0.27 ± 0.11	0.94 ± 0.05		
Consumed glycerol (mM)^*3^	192.7 ± 5.0	193.2 ± 2.4	168.2 ± 24.4	119.0 ± 44.6	117.6 ± 3.9		
Biomass (C-mol%)^*3^	37.0 ± 3.5	36.1 ± 1.4	19.6 ± 3.2	21.7 ± 11.0	20.8 ± 1.2	47.7	22.8
	(5.2 ± 0.5)	(5.1 ± 0.2)	(2.4 ± 0.6)	(1.6 ± 0.3)	(1.8 ± 0.1)		
3HP (C-mol%)^*3^	4.6 ± 0.8	5.7 ± 0.6	14.7 ± 7.0	20.1 ± 9.2	33.9 ± 1.2	0	70.5
	(8.9 ± 1.3)	(11.1 ± 1.0)	(24.3 ± 11.7)	(21.2 ± 7.7)	(39.9 ± 2.4)		
1,3-PDO (C-mol%)^*3^	0.8 ± 0.4	0.7 ± 0.2	22.9 ± 5.2	37.7 ± 13.2	5.9 ± 0.5	0	0
	(1.5 ± 0.8)	(1.3 ± 0.3)	(38.1 ± 9.4)	(40.5 ± 14.0)	(7.0 ± 0.7)		
Succinate (C-mol%)^*3^	0.9 ± 0.1	1.3 ± 0.2	0.3 ± 0.3	0	0	0	0
	(1.3 ± 0.2)	(1.9 ± 0.2)	(0.4 ± 0.3)	(0)	(0)		
Acetate (C-mol%)^*3^	9.9 ± 1.8	12.9 ± 0.2	8.2 ± 9.9	2.2 ± 4.1	6.6 ± 1.9	27.4	0
	(28.7 ± 5.4)	(37.3 ± 1.1)	(20.5 ± 24.3)	(5.1 ± 9.6)	(11.6 ± 3.0)		
Maximum methylglyoxal concentration (mM)	0.03 ± 0.02	0.03 ± 0.02	0.29 ± 0.02	0.22 ± 0.02	0.60 ± 0.02		

^*1^Specific growth rates were calculated using OD_600_ at 0–6 h for the TK52 and TK52z strains. For the TK52t, TK52tz and TK52tzy strains, specific growth rates during the 1st and 2nd growth phases were calculated using the OD_600_ at 0–6 h and 48–72 h, respectively.

^*2^Maximum 3HP production rates were calculated from the data at 24–48 h for the TK52 and TK52z strains, and at 48–72 h for other strains.

^*3^C-mol% was calculated from the carbon-mol of the product per carbon-mol of the consumed glycerol. The values in the parentheses indicate the final concentrations of biomass (g/L) and products (mM). Standard deviations were obtained from triplicate experiments in the TK52 and TK52z strains, and from 9 replicate experiments in other strains. For calculation of biomass yield, OD_600_ was converted into dry cell weight using the conversion factor 0.37 g DC/L, and carbon-mol in the biomass was calculated based on the biomass composition described in the iAF1260 model [21].

^*4^Metabolic simulation was performed with following parameters: GUR was 15 mmol/(g DC●h) and OUR was 10 mmol/(g DC●h).

### Gene knockout simulation for 3HP production

Metabolic simulation was carried out to improve 3HP production by considering the whole metabolic network. A genome-scale metabolic model of *E. coli*, iAF1260, which includes 2,077 metabolic and transport reactions and 1,038 unique metabolites [21], was employed with FBA [24,25] to simulate 3HP production in *E. coli*. Since the 3HP biosynthesis pathway does not exist in *E. coli*, seven reactions involved in the 3HP biosynthetic pathway were added to the iAF1260 model (Table 2), which was subsequently referred to as the iAF1260-3HP model. Using the iAF1260-3HP model, multiple gene knockout simulations were performed to identify candidate genes that could enhance 3HP production when deleted. For simulation parameters, glycerol was used as the sole carbon source, and its uptake rate (GUR) was set to 15 mmol/(g dry cell (DC)●h). The oxygen uptake rate (OUR) was set to 10 mmol/(g DC●h), which corresponds to a microaerobic condition.

**Table 2:** The metabolic reactions added to the iAF1260 model

**Reaction name**	**Metabolic reaction**	**EC number**
Glycerol dehydratase	Glycerol → 3HPA + H_2_O	4.2.1.30
3HPA dehydrogenase	3HPA + NAD^+^ + H_2_O → 3HP + NADH + 2H^+^	1.2.1.3
3HP transporter	3HP + H^+^ + → 3HP[e] + H^+^[e]	–
3HP exchange	3HP[e] →	–
1,3-PDO oxidoreductase	3HPA + NADPH + H^+^ → 1,3PDO + NADP^+^	1.1.1.202
1,3-PDO transporter	1,3-PDO → 1,3-PDO[e]	–
1,3-PDO exchange	1,3-PDO[e] →	–

Metabolites with “[e]” indicate extracellular metabolites.

In single-gene knockout simulations, solutions in which the metabolic flux of 3HP production was higher than zero were not obtained. Double-gene knockout simulations identified some candidate genes that when deleted together could enhance 3HP production (Table 3). Among these, Δ*tpiA* Δ*pgi*, Δ*tpiA* Δ*zwf*, and Δ*tpiA* Δ*edd* models displayed the highest carbon-mol yield of 3HP on glycerol (70.5 C-mol%). In this study, we focused on the double knockout of *tpiA* and *zwf* for further analysis. *tpiA* encodes for triosephosphate isomerase, which converts dihydroxyacetone phosphate (DHAP) to glyceraldehyde-3-phosphate (GAP). *zwf* codes for glucose-6-phosphate-1-dehydrogenase, which converts glucose-6-phosphate to 6-phospho-glucono-1,5-lactone.

**Table 3:** Knockout candidate genes for enhancing 3HP production obtained by gene knockout simulation

**Knockout genes**	**3HP yield (C-mol%)**	**Specific growth rate relative to wild-type**
iAF1260-3HP	0	100%
Δ*tpiA* Δ*zwf*	70.5	48%
Δ*tpiA* Δ*pgi*	70.5	48%
Δ*tpiA* Δ*edd*	70.5	48%
Δ*tpiA* Δ*gldA*	69.0	47%
Δ*tpiA* Δ*fsaB*	69.0	47%

**Figure 2:**
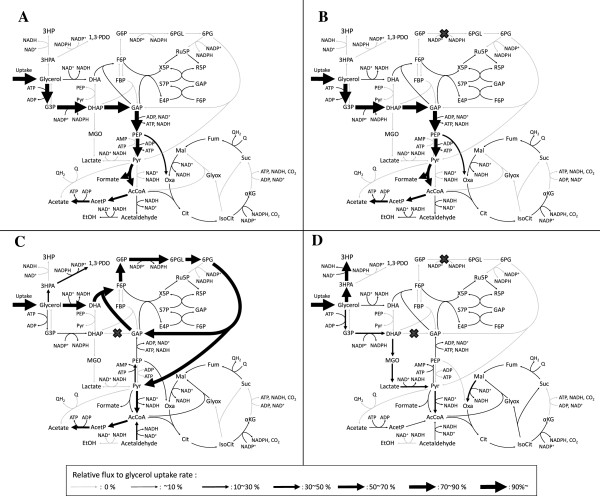
**Metabolic flux distributions based on the genome-scale metabolic model.** Metabolic flux distribution for the iAF1260-3HP **(A)**, Δ*zwf***(B)**, Δ*tpiA***(C)**, and Δ*tpiA* Δ*zwf***(D)** models are shown. Flux values are normalized by the glycerol uptake rate to 100%. Width of the black arrow corresponds to the relative flux value of glycerol uptake rate. Gray arrow indicates the flux of the corresponding reaction was 0. Abbreviations: 3HP, 3-hydroxypropionic acid; 3HPA, 3-hydroxypropionaldehyde; 1,3-PDO, 1,3-propanediol; DHA, dihydroxyacetone; DHAP, dihydroxyacetone phosphate; G3P, glycerol 3-phosphate; G6P, glucose 6-phosphate; 6PG, 6-phospho-gluconate; 6PGL, 6-phospho-glucono-1,5-lactone; F6P, fructose 6-phosphate; FBP, fructose 1,6-bisphosphate; Ru5P, ribulose 5-phosphate; R5P, ribose 5-phosphate; S7P, sedoheptulose 7-phosphate; GAP, glyceraldehyde 3-phosphate; X5P, xylulose 5-phosphate; E4P, erythrose4-phosphate; MGO, methylglyoxal; PEP, phosphoenolpyruvate; Pyr, pyruvate; AcCoA, acetyl-CoA; AcetP, acetyl phosphate; EtOH, ethanol; Oxa, oxaloacetate; Cit, citrate; IsoCit, isocitrate; α-KG, α-ketoglutarate; Suc, succinate; Fum, fumarate; Mal, malate; Glyox, glyoxylate; Q, ubiquinone; QH_2_, ubiquinol.

The simulated flux distributions of the iAF1260-3HP, Δ*tpiA*, Δ*zwf*, and Δ*tpiA* Δ*zwf* models are shown in Figure 2. In the iAF1260-3HP model, a high flux of glycolysis was predicted and ATP required for cell growth was mainly produced by glycolysis and the respiratory chain (Figure 2A). The Δ*zwf* model exhibited the same flux distribution as the iAF1260-3HP model (Figure 2B) because the flux of the glucose-6-phosphate-1-dehydrogenase reaction was zero in the iAF1260-3HP model (Figure 2A). In the Δ*tpiA* model, glycerol was mainly catabolized through the Entner-Doudoroff pathway via dihydroxyacetone (DHA) and entered into gluconeogenesis and the TCA cycle. The flux into glycolysis was blocked by the inability to generate GAP from DHAP because the amount of GAP consumed by the fructose 6-phosphate aldolase reaction, which converts DHA and GAP to fructose 6-phosphate, was equal to the amount of GAP produced by the Entner-Doudoroff pathway (Figure 2C).

In the Δ*tpiA* Δ*zwf* model, the *zwf* knockout blocked flux into the Entner-Doudoroff pathway, which was active in the Δ*tpiA* model. This resulted in glycerol catabolism through the glycerol kinase reaction, which converts glycerol to glycerol-3-phosphate, and the methylglyoxal pathway, in which DHAP is converted to pyruvate via methylglyoxal. 3HP was produced instead of acetate in the Δ*tpiA* Δ*zwf* model. In the Δ*tpiA* model, acetate production was preferred since ATP was also generated. However, in the Δ*tpiA* Δ*zwf* model, ATP was consumed by the glycerol kinase reaction, which is why total ATP was not generated by acetate production from glycerol. When the OUR was limited, 3HP production was increased, instead of acetate production, to balance the reduced capacity of the respiratory chain due to the reduction in NADH generation by 3HP production from glycerol (Figure 2D).

### 3HP production based on the metabolic simulation

Based on the results from the metabolic simulation, both *tpiA* and *zwf* were disrupted in the TK52 strain, generating strain TK52tz. The 3HP yield of TK52tz was successfully increased 4.4-fold relative to TK52 (20.1 ± 9.2 C-mol%) (Figure 1G-H and Table 1). TK52tz exhibited a two-step growth phase that was not observed in TK52, with specific growth rates of 0.54 1/h in the first growth phase (0–6 h) and 0.03 1/h in the second growth phase (48–72 h). 3HP was mainly produced in the second growth phase with the consumption of acetate and lactate that was produced prior to the second growth phase. The growth rate of TK52tz was decreased compared to TK52 (0.73 1/h), and glycerol was not completely consumed in TK52tz. Acetate and 1,3-PDO were produced as byproducts. Although 1,3-PDO production was not predicted by metabolic simulation, the experimental results of TK52tz were consistent with the simulation results, including the improvement in 3HP yield, the decrease in growth rate, and the lack of succinate production. This indicated that metabolic modeling is an effective strategy for the improvement of target production.

Based on the metabolic simulation, glycerol was predicted to be catabolized in TK52tz through the methylglyoxal pathway, which converts DHAP to pyruvate. This pathway is not usually active in *E. coli* due to allosteric inhibition by inorganic pyrophosphate and the low activity of enzymes involved in this pathway [37,38]. The extracellular concentration of methylglyoxal, an intermediate of the pathway, was increased significantly in TK52tz (0.22 mM at maximum) compared with TK52 (0.03 mM at maximum). This suggested that flux into the methylglyoxal pathway was increased as predicted, and this pathway might be a bottleneck for glycerol catabolism. This could result in decreased growth and incomplete glycerol consumption because methylglyoxal is a toxic cellular electrophile that reacts with the nucleophilic centers of macromolecules such as DNA, RNA, and protein [39].

Abundant production of 1,3-PDO was observed as a primary byproduct (37.7 ± 13.2 C-mol%) in TK52tz. Increased 3HP production might lead to the accumulation of intracellular 3HPA, a precursor of 3HP as well as 1,3-PDO, and result in the overflow of flux toward 1,3-PDO production. We speculated that changing the aldehyde dehydrogenase to the superior enzyme [17], or disruption of *yqhD*, whose product converts 3HPA to 1,3-PDO, would increase 3HP production and decrease 1,3-PDO production.

The single knockout strains of *tpiA* and *zwf*, TK52t and TK52z, respectively, were constructed to analyze the effects of the knockout of each gene. Metabolic simulation predicted that the *zwf* knockout would not affect metabolism, but the *tpiA* knockout would alter metabolic flux into the Entner-Doudoroff pathway and decrease the growth rate. Similar culture results between TK52z and TK52 (Figure 1A–1D, Table 1) suggest that the flux into the oxidative pentose phosphate pathway was small, and the deletion of *zwf* had a small impact on metabolism in this condition, as predicted by the simulation. The culture results of TK52t and TK52tz were similar (Figure 1E-1H, Table 1). The 3HP yield of TK52t was increased by 3.2-fold relative to TK52 (14.7 ± 7.0 C-mol%) and slightly reduced when compared to TK52tz. TK52t produced 1,3-PDO (22.9 ± 5.2 C-mol%) as a byproduct, as predicted by metabolic simulation. Acetate production by the *tpiA* knockout was also predicted, however measurements of acetate production (8.2 ± 9.9 C-mol%) in TK52t contained significant variation, thus it was difficult to compare the experimental results with the results from the metabolic simulation.

The increased 3HP yield in the *tpiA* knockout, which was not predicted by metabolic simulation, might be due to the conversion of glycerol to glycerol 3-phosphate by glycerol kinase, which may have had a higher activity than the glycerol dehydrogenase that converts glycerol to DHA [40]. This would lead to increased flux into the methylglyoxal pathway, as indicated by elevated methylglyoxal levels (Figure 1F), resulting in similar culture results for TK52tz (Figure 1H), such as 3HP production.

### Further increase in 3HP production by yqhD deletion, based on measurement of 1,3-PDO production

3HP production was successfully increased in TK52tz, however a high yield of 1,3-PDO was also produced (37.7 ± 13.2 C-mol%). *yqhD*, which is involved in 1,3-PDO biosynthesis, was deleted in TK52tz, generating the strain TK52tzy, in order to decrease 1,3-PDO production and further increase 3HP production. As a result, the 3HP yield of TK52tzy was increased 1.7-fold (33.9 ± 1.2 C-mol%) and the 1,3-PDO yield was drastically reduced (5.9 ± 0.5 C-mol%) relative to TK52tz (Figure 1I-J, Table 1). Compared to the parental strain, a 7.4-fold increase in 3HP yield was achieved in TK52tzy. Despite the deletion of *yqhD*, 1,3-PDO was still produced in TK52tzy (5.9 ± 0.5 C-mol%), likely due to the presence of other alcohol dehydrogenases that might convert 3HPA to 1,3-PDO. The deletion of *yqhD* increased the maximum concentration of extracellular methylglyoxal in TK52tzy (0.60 mM at maximum) relative to TK52tz (0.22 mM at maximum), since YqhD also utilizes methylglyoxal as a substrate [41]. The other culture results of TK52tzy, such as consumed glycerol and biomass yield, were similar to those of TK52tz (Table 1). The culture results of TK52t and TK52tz revealed large variations in the production of 3HP, 1,3-PDO, and acetate and the consumption of glycerol (Figure 1E-H). On the other hand, the results from TK52tzy displayed smaller variations in these measurements (Figure 1I-J). Furthermore, TK52t and TK52tz produced a large amount of 1,3-PDO, which accompanies NADPH oxidation. The deletion of *yqhD* in TK52tz decreased the magnitude of the error, suggesting that the high production of 1,3-PDO in TK52t and TK52tz might cause redox imbalance, resulting in the large variations in the measurements.

The 3HP yield of TK52tzy (33.9 ± 1.2 C-mol%) was comparable to previous studies producing 3HP from glycerol via flask cultivation. Mohan et al. achieved 3HP yield of 39.0 ± 0.01 C-mol% by the optimization of culture conditions such as the initial culture medium pH [18]. Rathnasingh et al. achieved a yield of 40 C-mol% by the expression of α-ketoglutaric semialdehyde dehydrogenase instead of aldehyde dehydrogenase, and periodic supplementation with vitamin B_12_, a coenzyme for glycerol dehydratase [17]. Jung et al. constructed an engineered *E. coli* strain by knocking out *ackA, pta*, and *yqhD* to reduce byproduct generation and knocking out *glpR* and overexpressing *glpF* to enhance glycerol metabolism [42]. They achieved high 3HP production (42 g/L) by fed-batch cultivation using a jar fermenter, but the 3HP yield was lower (26.2 C-mol%) than that achieved in this study. In previous studies [17,18], acetate was a major byproduct, as it was in this study, and higher yields of succinate, lactate, and ethanol were also produced. Succinate, lactate, and ethanol production might serve to oxidize the excess NADH that accompanies 3HP production and glycerol catabolism via glycolysis, since production of these metabolites requires NADH as a reducing agent. Conversely, the yields of these metabolites were small in TK52tzy. This might be because the deletion of *tpiA* and *zwf* prevented flux into glycolysis, reducing excess NADH production, as predicted by metabolic simulation.

In this study, metabolic simulation was performed under the conditions of OUR/GUR = 0.67 (OUR = 10 mmol/(g DC●h) and GUR = 15 mmol/(g DC●h)), and additional simulations were also performed under other various OUR/GUR conditions (data not shown). Given the 3HP yield of TK52tzy (33.9 ± 1.2 C-mol%), the metabolic state of the cell in this study was estimated under the condition of OUR/GUR ≈ 1, by the metabolic simulation. Although the adjustment of oxygen supply to the simulation result was difficult using flask cultivation (oxygen transfer coefficient, k_L_a, of a shaking flask is 10–100 1/h), the metabolic simulation was used effectively for improving 3HP production. Further increases in 3HP production can be expected by the optimization of aeration conditions using a bioreactor.

Metabolic simulation is a powerful tool for the design of metabolic engineering strategies to improve target production and has been used successfully in many studies. Metabolic simulation using FBA is simply based on the assumption of steady state metabolism without considering the complex cellular mechanisms such as enzyme activity and regulation. This can cause discrepancies between the simulation and experimental results, i.e., the unpredicted production of 1,3-PDO in the present study. Thus, examination of the differences between experimental and simulation results, and conformation of the constructed strains to the simulated metabolic state, is important for further improvement of target production.

## Conclusions

In conclusion, the production of 3HP from glycerol in *E. coli* was improved by the modification of central metabolism, based on metabolic simulation and experimental validation of the engineered strains. Gene knockout simulations using the genome-scale metabolic model identified *tpiA* and *zwf* as candidates to be modified and in this double knockout strain, TK52tz, 3HP yield was increased 4.4-fold relative to the TK52 parent strain, as predicted. Increased extracellular methylglyoxal in TK52tz suggested that glycerol catabolism through the methylglyoxal pathway was consistent with metabolic simulation. Since TK52tz produced 1,3-PDO as an abundant byproduct, *yqhD*, which encodes an enzyme involved in 1,3-PDO production, was deleted in TK52tz. The resulting strain, TK52tzy, exhibited a 1.7-fold increase in 3HP yield relative to TK52tz and a 7.4-fold increase (33.9 ± 1.2 C-mol%) relative to TK52. The double knockout of *tpiA* and *zwf* contributed to the reduced production of byproducts such as succinate and lactate that are associated with NADH oxidation, likely due to reduced NADH production by the inhibition of glycolysis. The successful increase in 3HP production, based on metabolic simulation, demonstrated the effectiveness of metabolic modeling in designing a metabolic engineering strategy. Moreover, experimental validation of the engineered strains and comparison with the simulation results provided additional modifications to the engineering strategy to increase target production.

## Methods

### Strains and plasmids

The strains used in this study are summarized in Table 4. The MG1655(DE3) strain was constructed based on *E. coli* MG1655, using the λDE3 Lysogenization Kit (Merck KGaA, Darmstadt, Germany). The 3HP-producing strain, TK52, was constructed as published previously [17]. Fragments containing *dhaB1*, *dhaB2*, and *dhaB3*, encoding for components of glycerol dehydratase, and the *gdrA* and *gdrB* genes, encoding for glycerol dehydratase reactivase, were amplified from the genomic DNA of *K. pneumoniae* subsp. *pneumoniae* (NBRC 14940), which was purchased from the National Institute of Technology and Evaluation (Tokyo, Japan). Fragments were generated by PCR using the primer pair 5′-CCGGAATTCATGAAAAGATCAAAACGATTTGCAGTACT-3′ and 5′-GTTAAGCTTGATCTCCCACTGACCAAAGCTGG-3′ for *dhaB* (*dhaB1*, *dhaB2*, *dhaB3*) and *gdrA* and the primer pair 5′-GAAAAGCTTGAGGGGGACCGTCATGTCGCTTTCACCGCCAG-3′ and 5′-GCGCTTAAGTCAGTTTCTCTCACTTAACGGC-3′ for *gdrB*. The *aldH* gene was amplified from the genomic DNA of *E. coli* MG1655 with the primer pair 5′-GGAGGATCCATGAATTTTCATCATCTGGC-3′ and 5′-TCGAAGCTTTCAGGCCTCCAGGCTTAT-3′. PCR was performed using KOD FX Neo (Toyobo Co., Ltd., Osaka, Japan). Each amplified fragment was treated with A-attachment mix (Toyobo Co., Ltd.), and then cloned into pGEM-T easy (Promega Co., Madison, WI, U.S.A.), followed by sequence confirmation by the BigDye Terminator v3.1 Cycle Sequencing Kit (Applied Biosystems, Inc., Foster City, CA, U.S.A.), and the 3130 Genetic Analyzer (Applied Biosystems). The *Eco*RI-*Hin*dIII and *Hin*dIII-*Afl*II fragments carrying *dhaB-gdrA* and *gdrB*, respectively, on pGEM-T easy were cloned into the same restriction sites in pCDFDuet-1 (Merck KGaA), generating pCDFDuet-1/*dhaB*-*gdrAB*. In addition, the *Bam*HI-*Hin*dIII fragment carrying *aldH* on pGEM-T easy was cloned into the pTrc99A expression vector (Pharmacia, Stockholm, Sweden), generating pTrc99A/*aldH*.

**Table 4:** **The *E. coli *** **strains used in this study**

**Strain**	**Genotype**	**Source**
BW25113	F^−^, λ^−^, *lacI*^q^*rrnB*_T14_ Δ*lacZ*_WJ16_*hsdR514* Δ*araBA-D*_AH33_ Δ*rhaBAD*_LD78_	Datsenko and Wanner [43]
JW3890	The same as BW25113 but Δ*tpiA*::*kan*	Baba *et al.*[44]
BW25113 Δ *zwf*::*cat*	The same as BW25113 but Δ*zwf*::*cat*	Nakahigashi *et al.*[45]
BW25113 Δ *yqhD*::*tet*	The same as BW25113 but Δ*yqhD*::*tet*	This study
MG1655(DE3)	F^−^, λ^−^, *rph-1,* λ (DE3[*lacI lacUV*5-*T*7 *gene* l *indl sam*7 *nin5*])	This study
TK52	MG1655(DE3) transformed with pTrc99A/*aldH* and pCDFDuet-1/*dhaB*-*gdrAB*	This study
TK52t	The same as TK52 but Δ*tpi::kan*	This study
TK52z	The same as TK52 but Δ*zwf::cat*	This study
TK52tz	The same as TK52 but Δ*tpiA::kan* Δ*zwf::cat*	This study
TK52tzy	The same as TK52 but Δ*tpiA::kan* Δ*zwf::cat* Δ*yqhD::tet*	This study

The knockout strains were constructed using Wanner’s method [43] and P1*kc*-mediated phage transduction [46]. For the deletion of *yqhD*, the disruption cassette, including the tetracycline resistance gene and homologous regions upstream and downstream of *yqhD*, was amplified from pKD13tet [45] by PCR with the primer pair 5′-GCAGATCGTTCTCTGCCCTCATATTGGCCCAGCAAAGGGAGCAAGTAATGATTCCGGGGATCCGTCGACC-3′ and 5′-CGAAAACGAAAGTTTGAGGCGTAAAAAGCTTAGCGGGCGGCTTCGTATATTGTAGGCTGGAGCTGCTTCG-3′. The disruption cassette was introduced into the BW25113/pKD46 strain to construct BW25113Δ*yqhD*::*tet*. To delete *zwf*, *tpiA*, and *yqhD* in *E. coli* MG1655(DE3), P1 transduction was performed using P1 phage obtained from JW3890 [44], BW25113Δ*zwf*::*cat* and BW25113Δ*yqhD*::*tet* strains, respectively. Finally, the plasmids pTrc99A/*aldH* and pCDFDuet-1/*dhaB*-*gdrAB*, were introduced into MG1655(DE3) and the knockout strains to construct the 3HP-producing strains (Table 4).

### Medium and culture methods

Pre-cultures were grown aerobically at 37°C overnight in Lennox medium (10 g/L tryptone, 5 g/L yeast extract, 5 g/L NaCl, 1 g/L glucose) containing 0.05 g/L ampicillin and 0.05 g/L streptomycin. Pre-cultures were transferred to the main culture with an initial optical density at 600 nm (OD_600_) of 0.02. For the main culture, M9 medium (17.1 g/L Na_2_HPO_4_･12H_2_O, 3.0 g/L KH_2_PO_4_, 2.0 g/L NH_4_Cl, 0.5 g/L NaCl, 0.123 g/L MgSO_4_･7H_2_O, 0.00278 g/L FeSO_4_･7H_2_O, 0.0147 g/L CaCl_2_･2H_2_O, 0.01 g/L thiamine HCl) supplemented with 2 g/L yeast extract, 0.02 mM cyanocobalamin, 0.1 mM IPTG, 0.05 g/L ampicillin, and 0.05 g/L streptomycin was used. Cells were cultured in 500 mL Sakaguchi flasks containing 50 mL of the M9 medium at 37°C in a shaking incubator at 120 rpm (MM-10, Taitec, Saitama, Japan).

### Analytical methods

Cell growth was monitored by the measurement of OD_600_ using UV-mini 1240 (Shimadzu, Kyoto, Japan). Concentrations of glycerol, 3HP, 1,3-PDO, succinate, lactate, acetate, formate, and ethanol present in the supernatant of the culture broth were determined by an HPLC system (HPLC Prominence, Shimadzu) equipped with an Aminex HPX-87H column (Bio-Rad, Hercules, CA, U.S.A.), a UV/vis detector (SPD-20A), and a refractive index detector (RID-10A). The column temperature was set to 65°C, and 2 mM H_2_SO_4_ was used as the mobile phase with a flow rate of 0.5 mL/min. The flow cell temperature of the refractive index detector was set to 35°C. The supernatant of the culture broth was obtained by centrifugation at 21,500 × *g* for 5 min at 4°C, and then filtered through a Millex HV 0.45-μm filter (Merck KGaA).

Methylglyoxal in the supernatant was quantified colorimetrically with 2,4-dinitrophenylhydrazine (2,4-DNPH) [47]. The reaction mixture containing 67 μL of sample and 22 μL of 2,4-DNPH solution (0.1% 2,4-DNPH in 2 M HCl) was incubated for 15 min at 30°C in a 96-well microtiter plate, and then 111 μL of 10% NaOH was added. After a 15-min incubation at room temperature, the absorbance at 544 nm was measured with a microplate reader (1420 ARVO, PerkinElmer Inc., Waltham, MA, U.S.A.).

### In silico metabolic simulation

The genome-scale metabolic model of *E. coli* K-12, named iAF1260 [21], was used. iAF1260 contains 1,260 ORFs, 2,077 metabolic and transport reactions, and 1,038 unique metabolites. To simulate 3HP production by *E. coli*, seven reactions required for 3HP biosynthesis from glycerol were added to the iAF1260 model (Table 2), because neither the iAF1260 model nor the wildtype *E. coli* contained the 3HP biosynthesis pathway. The 3HP transport reaction was also added to the metabolic model as a proton symporter because the transport of a similar metabolite, D-lactate (2-hydroxypropionic acid), occurs through a proton symporter in the iAF1260 model. In the same manner, the 1,3-PDO transport reaction was added as a diffusion reaction, which is considered to utilize the 1,2-propandiol transporter in the iAF1260 model.

FBA was used to simulate the metabolic flux distribution in the genome-scale metabolic model [24,25]. Briefly, a pseudo-steady state of the metabolite profile was assumed, and the maximum and minimum ranges of the flux for each reaction were defined. These constraints provided a feasible space for the flux distribution in the metabolic model. To obtain an optimal flux distribution in the feasible space, an objective function was introduced and a linear programming technique was applied. This problem is represented by the following equation:

$$\max\mathrm{imize}:c^{T}\cdot v$$

subjectto:⋅S⋅v=0

$$v_{\min}\leq v\leq v_{\max}$$

where *S* represents the stoichiometric matrix of metabolites in metabolic reactions, and *v* indicates a vector of flux for each metabolic reaction. The values *v*_min_ and *v*_max_ represent the minimum and maximum constraints of flux for each reaction, and *c* is a vector that represents the objective function to be maximized or minimized. In this study, biomass production was used as the objective function to be maximized, with the assumption that cellular metabolism is self-organized to maximize growth rate. After the maximal biomass production flux was obtained, the 3HP production flux was maximized under the fixed biomass production flux, on the maximal value, to avoid undetermined 3HP production flux [29].

For metabolic simulation, glycerol was used as the sole carbon source, and the uptake rate was set to 15 mmol/(g DC●h). The oxygen uptake rate was set to 10 mmol/(g DC●h). Other external metabolites such as CO_2_ and NH_3_ were allowed to transport freely through the cell membrane. For gene knockout simulations, minimum and maximum fluxes of the corresponding reactions were set to zero. All simulations were performed using Matlab (MathWorks Inc., Natick, MA, U.S.A.) with a solver for linear programming, GLPK (GNU Linear Programming Kit, http://glpkmex.sourceforge.net/).

## Abbreviations

3HP: 3-hydroxypropionic acid; FBA: Flux balance analysis; 1,3-propanediol: 1,3-PDO; 3HPA: 3-hydroxypropionaldehyde; GUR: Glycerol uptake rate; DC: Dry cell; OUR: Oxygen uptake rate; C-mol%: Carbon-molar yield; DHAP: Dihydroxyacetone phosphate; GAP: Glyceraldehyde-3-phosphate; DHA: Dihydroxyacetone; 2,4-DNPH: 2,4-dinitrophenylhydrazine; G3P: Glycerol 3-phosphate; G6P: Glucose 6-phosphate; 6PG: 6-phospho-gluconate; 6PGL: 6-phospho-glucono-1,5-lactone; F6P: Fructose 6-phosphate; FBP: Fructose 1,6-bisphosphate; Ru5P: Ribulose 5-phosphate; R5P: Ribose 5-phosphate; S7P: Sedoheptulose 7-phosphate; X5P: Xylulose 5-phosphate; E4P: Erythrose 4-phosphate; MGO: Methylglyoxal; PEP: Phosphoenolpyruvate; Pyr: Pyruvate; AcCoA: Acetyl-CoA; AcetP: Acetyl phosphate; EtOH: Ethanol; Oxa: Oxaloacetate; Cit: Citrate; IsoCit: Isocitrate; α-KG: α-ketoglutarate; Suc: Succinate; Fum: Fumarate; Mal: Malate; Glyox: Glyoxylate; Q: Ubiquinone; QH2: Ubiquinol.

## Competing interests

The authors have no conflicts of interest to declare.

## Authors’ contributions

KT carried out the strain construction and the culture experiments and drafted the manuscript. SO performed the metabolic simulation. KY participated in the design of the study, and drafted the manuscript. TH participated in the design of the study. ST carried out the strain construction. CF participated in the design of the study. HS conceived and supervised the study. All authors read and approved the final manuscript.
